# Middle Ear Neuroendocrine Adenoma: A Case Report and Literature Review

**DOI:** 10.1155/2020/8863188

**Published:** 2020-12-21

**Authors:** Luca Bruschini, Rachele Canelli, Christina Cambi, Giacomo Fiacchini, Stefano Berrettini, Francesca Forli

**Affiliations:** ENT Audiology and Phoniatric Unit, University Hospital of Pisa, Via Paradisa 2, Pisa 56100, Italy

## Abstract

Neuroendocrine adenomas of the middle ear are rare tumors that represent less than 2% of primary tumors of the ear. In this paper, we describe a case of a 40-year-old woman who developed neuroendocrine adenoma of the middle ear. The specific management strategy for this rare tumor is unclear; information in the available literature on the management of this tumor is varied. However, an extensive demolition seems to be the gold standard treatment for this tumor to avoid recurrence and regional metastases in the lymph node or distant metastases. For the present case, we performed an incisional biopsy to confirm the diagnosis, and thereafter, we performed a canal-wall-down tympanoplasty. For cases like the present one, careful long-term clinical and instrumental follow-up is required to monitor progress and facilitate patient recovery.

## 1. Introduction

Neuroendocrine adenoma of the middle ear was described for the first time in 1976 [1]. These tumors make up less than 2% of primary tumors of the ear [2]. The classification, histogenesis, and biologic behavior of this tumor have been reported in the literature in various ways; the tumor has been inappropriately described as a ceruminoma, a ceruminous adenoma, an adenomatous tumor, an adenocarcinoid, and an amphicrine tumor [1–7].

Neuroendocrine adenomas show a low aggressive biological behavior with slow local growth, but they can recur and cause local and distant metastases, suggesting they should be classified as having low-grade malignancy [2]. Reaching a definite clinical diagnosis is challenging, and an immunohistochemical investigation is often required [3]. This rare tumor does not show a predilection for sex or age of onset [4].

Herein, we report a case of a 40-year-old woman treated for a neuroendocrine adenoma in her right ear and present a brief review of the available literature on this rare tumor.

### 1.1. Case Presentation

A 40-year-old woman presented to our clinic in February 2019 with a four-year history of aural fullness in her right ear associated with recurrent otalgia, itching, headache, and postural instability. She did not have any other otologic and neurologic symptoms or signs.

In Oct 2015 and April 2018, the patient went to an emergency room because her headaches worsened during these periods. The headaches were cluster headaches characterized by severe pain in the frontal and temporal regions. In 2018, the headaches were associated with a mucopurulent otitis in her right ear. The patient underwent two brain computed tomography (CT) scans, one for each hospitalization. In 2018, the second CT scan revealed signs of inflammation of the hypotympanum and mesotympanum of her right ear. The patient was treated with antibiotic therapy.

**Figure 1:**
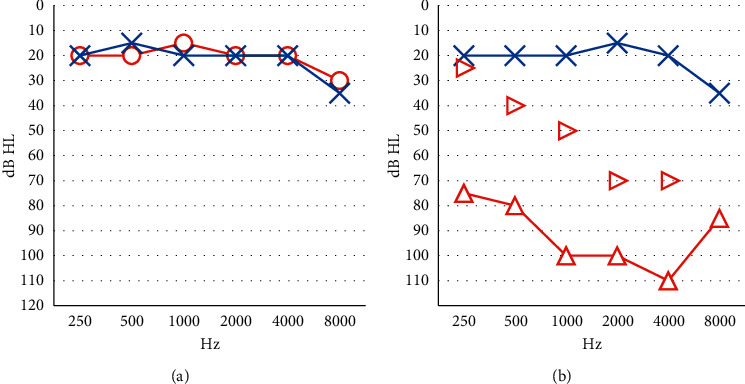
Audiometric threshold before (a) and after (b) the surgical treatment. Red circles and lines: right ear air threshold, red triangles and lines: masked right ear air threshold, red triangles: right ear bone threshold, and blue crosses and lines: left ear air threshold.

At the time of presentation to our clinic, she had no history of neoplastic pathologies, smoking, or alcoholism. Clinical examination showed that her right tympanic membrane was intact, but some whitish material could be seen at the retrotympanic level. Measurement of her audiometric threshold showed that she had bilateral normal hearing levels (Figure 1(a)).

**Figure 2:**
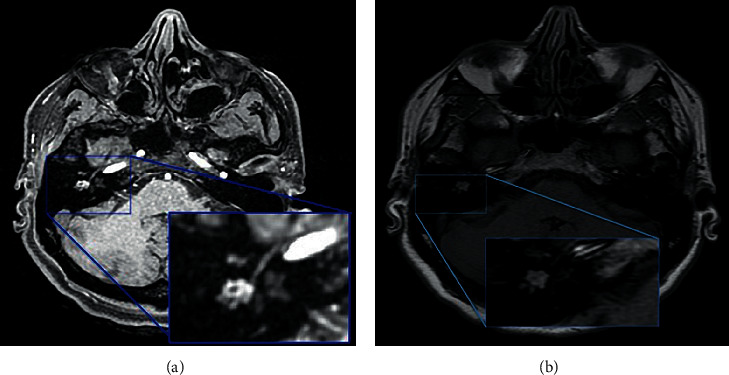
Axial magnetic resonance imaging (MRI) scans of the middle ear. (a) T1 fat-saturated MRI revealed tissue in the middle ear. (b) T1 diffusion-weighted imaging did not show a restriction of diffusion in the tissue, as is typical of cholesteatoma.

**Figure 3:**
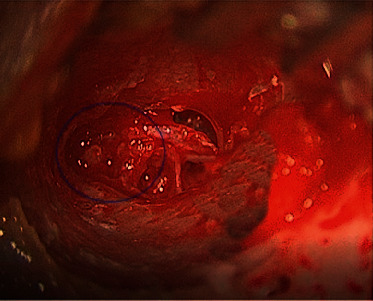
Neuroendocrine adenoma of the middle ear (encircled) during the first surgery; view from the ear canal.

**Figure 4:**
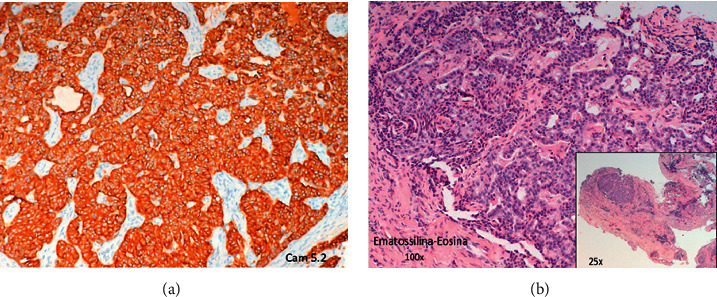
(a) Hematoxylin and eosin stain; the tumor was unencapsulated with an organoid growth pattern characterized by rounded nests and trabecular and glandular aggregates, with hyalinized fibrous stroma in the background (magnification ×25). Cytologically, the tumor cells lacked nuclear pleomorphism and exhibited finely dispersed nuclear chromatin (magnification ×100). (b) Immunohistochemistry results showed diffuse positivity for Cam 5.2.

Magnetic resonance imaging (MRI) of the ear did not reveal any signs of cholesteatoma (i.e., hyperintensity on diffusion-weighted imaging) in the right ear (Figure 2); therefore, we decided to proceed with an excisional biopsy to establish a diagnosis. During the surgical procedure, the lesion appeared as a reddish mass with no capsule and had a parenchymatous consistency (Figure 3). The ossicular chain was not interrupted, but it was enveloped by pathological tissue. There were no signs of erosion in the inner ear and the facial canal. The results of intraoperative histological examination were suggestive of paraganglioma, whereas a definitive histological report highlighted both the presence of neuroendocrine markers (synaptophysin and partial expression of chromogranin A) and epithelial markers (cytokeratin 7 and Cam 5.3) (Figure 4). Immunochemical investigation for the p63, CD56, and S100 markers returned negative results. The proliferation activity of Ki-67 was <3%. Given these findings, neuroendocrine adenoma of the middle ear was diagnosed.

**Figure 5:**
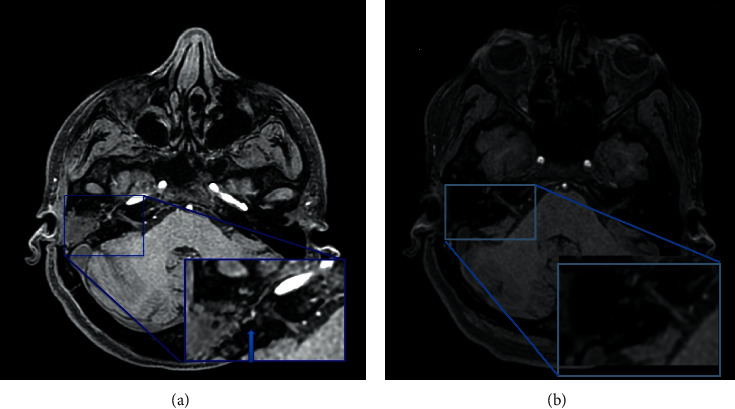
(a) Axial T1 fat-saturated magnetic resonance imaging scan of the middle ear; blue arrow indicated the residual tumor in the middle ear before the canal-wall-down tympanoplasty. (b) Axial T1 fat-saturated magnetic resonance imaging scan of the middle ear after one year from the surgical procedure; the blue rectangle showed the absence of recurrence or persistence of the disease.

After the diagnosis was made, examination using positron emission tomography/CT did not reveal any local or distant metastases. Therefore, a new MRI was performed to monitor the state of the local disease. The imaging showed some pathological residual tissue (Figure 5(a)).

A multidisciplinary team, which comprised a radiologist, a radiotherapist, an oncologist, and an otolaryngologist, determined surgical treatment to be the best therapeutic choice to treat the disease. A radical demolition to eradicate the residual disease was carried out by performing a canal-wall-down tympanoplasty removing the stapes and footplate and with preservation of the optic capsule. The patient was hospitalized for six days; this period included the duration of the surgery and the postoperative period.

Postoperative audiometric examination revealed the presence of good auditory residue in the operated ear (Figure 1(b)). The patient is currently disease-free and is undergoing follow-up at our clinic. As at the time of writing this paper, the follow-up duration was at 12 months. We performed an otoscopy on the patient every two months. A new MRI was performed at one year postoperatively, and it showed the absence of recurrence or persistence of the disease (Figure 5(b)). The patient was disease-free at one year from the surgical treatment.

Until today, the patient was not treated to restore the hearing loss. We retain to suggest bone-anchored hearing aids after 3–5 years from the treatment of the tumor.

## 2. Discussion

In this report, we present the details of a rare case of neuroendocrine adenoma of the middle ear. The pathological and immunohistochemical findings of this case were consistent with those described in the existing literature [4–7].

The pathogenesis of this tumor is currently a topic of discussion, with different authors proposing different origins. Hyams and Michaels speculated that it may originate from the cells of the mucosa of the middle ear [1]; they also suggested that it may originate from off-site embryonic nests of glandular cells in the middle ear mucosa [1]. Katabi and Torske and Thompson proposed that it may originate from undifferentiated pluripotential endodermal stem cells since epithelial cells with neuroendocrine differentiation do not exist in the middle ear [2, 6].

Patients with this tumor usually present with hearing loss, otalgia and ear fullness, and an intact eardrum behind a visible brown-red mass. A diagnosis is often delayed due to the rarity of this tumor, and biopsy is usually needed to reach a definite diagnosis. In most cases, such as in ours, cholesteatoma is the first suspect. The differential diagnoses of a neuroendocrine adenoma include jugulotympanic paraganglioma, vascular malformations, acoustic neurinoma, meningioma, endolymphatic sac papillary tumor, rhabdomyosarcoma, teratoid tumor, and adenocarcinoma [8–10]. However, histologic, imaging, and immunohistochemical investigations allow for an accurate diagnosis [2].

Several authors have proposed different classification systems because this neoplasm, although seldom, tends to exhibit local invasion, local recurrence, and metastatic potential [7, 8, 11]. Saliba and Evrard proposed that middle ear glandular neoplasms be classified into three types based on the expression of neuroendocrine markers and the presence of metastases [7]. Type I, which is the most common, is called neuroendocrine adenoma of the middle ear and has an incidence of 76%. It is characterized by the presence of immunohistochemical markers but no metastases. Type II is the middle ear adenoma, which represents 20% of cases with negative immunohistochemical markers and metastases. Type III is the least common; it makes up 4% of cases and is characterized by immunohistochemical markers and metastases [2, 7].

In the 2005 World Health Organization classification, these tumors were classified as neuroendocrine carcinomas with low-grade epithelial differentiation (grade I), whereas in the recent 2017 classification, they were classified as adenomas with neuroendocrine features [8].

Marinelli et al. in a multi-institutional retrospective study on 32 cases proposed a “T/N/M/S” staging system [11]. In accordance with this classification, the case of this report was T2bN0M0S0, i.e., it fills the middle ear and encloses the ossicles, extending into the mastoid, without regional or distant metastases, and it was a nonsecretory tumor. About one-third of T2 MEANTs and nearly two-thirds of T3 MEANTs developed a local recurrence after a medium follow-up time of 6 years [11].

Based on information in the existing literature, a complete surgical resection is the primary treatment method for neuroendocrine adenomas of the middle ear. Although four metastatic cases treated with adjuvant radiotherapy have been previously reported, chemotherapy has not been described as a viable treatment method [12, 13]. Local recurrence and metastasis were reported in three out of the four cases treated with radiotherapy, and the radiotherapy itself is suspected to induce a malignant transformation of the tumor [6, 12]. So, to eradicate all the tumors, we performed a canal-wall-down tympanoplasty, removing the stapes, a residual part of the disease. We did not propose postoperative radiotherapy and/or chemotherapy.

A middle ear adenomatous neuroendocrine tumor is a rare tumor that is characterized by an indolent course and an uncertain malignant potential. Due to its ability to recur locally, the regional and distant metastases reported in the available literature, and the absence of sufficiently valid data to predict the progress of this tumor, extensive demolition with negative margins and careful long-term clinical and instrumental follow-up are required to monitor its biological progress.
